# Serum vitamin D status in children with protein-energy malnutrition admitted to a national referral hospital in Uganda

**DOI:** 10.1186/s13104-015-1395-2

**Published:** 2015-09-07

**Authors:** Henry W. Nabeta, Josephine Kasolo, Reuben K. Kiggundu, Agnes N. Kiragga, Sarah Kiguli

**Affiliations:** Department of Physiology, College of Health Sciences, Makerere University, Kampala, Uganda; Infectious Diseases Institute, College of Health Sciences, Makerere University, Kampala, Uganda; Department of Pediatrics and Child Health, College of Health Sciences, Makerere University, Kampala, Uganda

**Keywords:** Vitamin D, Malnutrition, Children, Uganda

## Abstract

**Background:**

Vitamin D deficiency is a world-wide epidemic with recent estimates indicating that greater than 50 % of the global population is at risk. In Uganda, 80 % of healthy community children in a survey were found to be vitamin D insufficient. Protein-energy malnutrition is likely to be associated with vitamin D intake deficiency. The aim of this study was to determine the prevalence of vitamin D deficiency and the associated factors among children admitted with protein-energy malnutrition to the pediatrics wards of Mulago hospital in Kampala, Uganda.

**Methods:**

Consecutive sampling was done with 158 children, aged 6–24 months, enrolled in a cross sectional study. One hundred and seventeen malnourished and 41 non malnourished children were enrolled from the Acute Care unit, pediatrics in-patient wards, outpatient and immunization clinics, following informed consent obtained from the children’s parents/guardians. Children with protein energy malnutrition were categorized based on anthropometric measurements of weight-for-height and weight for length compared with the recommended WHO reference Z-score. Serum 25-hydroxyvitamin D, calcium and phosphate were assayed.

**Results:**

One hundred seventeen malnourished and 41 non malnourished children were enrolled. The majority of study participants were male, 91 (57.6 %). The mean serum vitamin D levels among the malnourished was 32.5 mmol/L (±12.0 SD) and 32.2 mmol/L (10.9 SD) among the malnourished, p = 0.868. Fifteen (36.6 %) of the non malnourished children and 51 (43.6 %) of the malnourished had suboptimal levels, p = 0.689. Malnourished children admitted with meningitis and cerebral palsy had lower serum vitamin D levels than those with other infections.

**Conclusion:**

There was no statistically significant difference in vitamin D values between the malnourished and non malnourished children. Clinicians should actively screen for children for serum vitamin D levels regardless of nutritional status.

## Background

Vitamin D deficiency is a world-wide epidemic [1] with recent estimates indicating greater than 50 % of the global population is at risk [2]. A high prevalence of vitamin D deficiency has been found across all age groups in all populations studied in countries around the globe [3]. It is estimated that 1 billion people worldwide have either vitamin D insufficiency or deficiency [4]. In tropical African countries the mean vitamin D values are high, but individuals with deficiencies have lower values than their peers. Collectively the prevalence averages between 5 and 20 % in most age groups [1].

Protein-energy malnutrition (PEM) is likely to be associated with vitamin D intake deficiency. Moreover, some individuals with seemingly adequate ultraviolet (UV) exposure have low serum vitamin D concentration, due to the varying levels of the skin pigmentation [5]. Hyperpigmentation in black people can compromise vitamin D production and this phenomenon may be aggravated by limited sun light exposure in the young infants [5].

PEM is globally the most important risk factor for illness and death, contributing to more than half of deaths in children worldwide, where child malnutrition was associated with 54 % of deaths in children in developing countries in 2001 [6]. PEM is still a major public health issue in developing countries [7]. It is associated with as much as 50–60 % of under-five mortality in poor countries [8, 9] and a myriad of morbidities [10]. According to the UNICEF estimates, the underweight rate of children in Uganda in 2006 was 16.4 % [11]. Some children attending Mulago hospital were found in poor nutrition state, suffering from kwashiorkor and marasmus with a good number being stunted [12].

Children that present to hospital may have underlying infections that can either contribute to or can be associated with low vitamin D levels. Gastrointestinal and ear infections have been associated with low serum vitamin D levels [12]. Vitamin D deficiency has been shown to be responsible for alterations in the immune response leading to an increased risk of infection. It has been postulated that these infections may predispose to vitamin D deficiency especially increased rates of diarrhea with vomiting and earache/discharge with fever. Celiac disease, associated with abdominal pain, diarrhea and weight loss, osteopenia or osteoporosis and osteomalacia have been found in vitamin D deficiency [13]. There has been mixed results in studies that have followed patients with malaria and assayed for vitamin D. Newens et al. [14] followed a group of patients admitted in hospital with *Plasmodium falciparum* and did not find a difference in serum 25(OH)D levels at different time points and at follow-up after discharge. Recent studies have postulated the role of vitamin D insufficiency in the development of severe malaria [15] and pneumonia. This may be due to the reduction in B cell and natural killer cell lines that was found in patients studied with pneumonia and nutritional rickets [16].

This study aimed at determining the prevalence of vitamin D deficiency and the associated factors among children admitted with protein-energy malnutrition to the pediatric wards of Mulago hospital in Kampala, Uganda.

## Methods

### Study design and participants

This was a cross sectional study conducted on the pediatrics in-patient and outpatient wards, acute care unit and immunization clinics of Mulago hospital in Uganda. Purposive sampling was employed. This hospital, found in Kampala, the capital city of Uganda, lies within the tropics and receives sunshine all the year round. The population served is both urban and peri-urban.

Children between the ages of 6–24 months, whose parents gave informed consent, were enrolled into the study from February 2011 to September 2013. Children with protein energy malnutrition were categorized based on anthropometric findings. This was assessed depending on the measurements of weight-for-height and weight for length. Malnourished children were those that were either classified as severe acute malnutrition (SAM) or moderate acute malnutrition (MAM) based on the WHO reference Z score. MAM were children below −2 and up to −3 SD. SAM were children below −3 SD. The non malnourished were children who were defined as normal by the Z score, above −1 SD.

Children taking anticonvulsants like phenobarbitone, vitamin D supplements, fortified formula milk, and those whose parents/guardians did not give informed consent were excluded from the study.

### Anthropometric assessment

#### Data collection

Questionnaires were administered to the parents/guardians of the enrolled children. The information collected included socio demographic data, medical and drug history, nutrition and breastfeeding history. A physical examination was then performed on the child.

Dietary history was assessed following a structured interview, where parents/guardians were asked to recall all food and drink during the previous 24 h prior to the child’s admission in hospital that the child had been able to take. Prompts were also made for the quantification of portion sizes that had been consumed. The breastfeeding history and normal daily diet was also probed from the children’s caretakers following questions adapted from the standardized NIH diet history questionnaire (DHQ). This tool has been found to perform well in assessing diet-disease risk [17].

Sunshine exposure was assessed by asking parents/guardians to estimate the average daily duration in hours that the child regularly spent outdoors (in the sunshine). These questions posed to the parents/guardians during the structured interview were adapted from the sun-exposure questionnaire [18]. The sun-exposure questionnaire was originally devised to assess exposure to sunlight over a season. The format required the parents/guardians to describe outdoor activities on a typical day and also atypical days when there may have been more or less outdoor activity than usual. Four non-consecutive 24-h recalls of daily activities and time spent outdoors were ascertained and the average period of exposure taken.

Weight was measured using the standardized scale balance (Salter-England Model 235 6S) for children who were able to stand, and the Infant Scale was used for those unable to stand. Height was measured using the standardized height scale (Secca^®^). Body length was measured using the length board (Shorr Board TM-USA), following standard procedures. Mid upper arm circumference was measured using the standard tape.

Blood was drawn from the enrolled participants for measurement of vitamin D, calcium and phosphate.

### Blood sample collection and laboratory analysis

A venous blood sample of 4–5 mL was drawn from either the antecubital or femoral vein using a needle and syringe, into a heparinized blood collection tube from each child. Blood was centrifuged, and the plasma extracted was stored at −20 °C. Vitamin D concentrations were determined by an immunoassay technique using the Elecsys vitamin D assay. This is an electro-chemilumniscense immunoassay supplied by Roche diagnostics, Germany, which measures the vitamin D concentrations in the range of 4–100 ng/mL [19]. Measurements were done using kits, calibrators and controls from the same manufacturer. Serum calcium and phosphate were analysed using a full automated COBAS 6000 (Roche Diagnostics GmbH, Germany) machine.

### Statistical analysis

Data entry and cleaning was done using Epidata version 3.02. Data was analyzed using statistical package, STATA Version 11.1. Baseline characteristics were compared against outcome using Chi square test. Continuous variables were compared using *t* test for normally distributed data and Wilcoxon rank sum test for data not normally distributed. Patient characteristics were reported as frequency and percentage for categorical variables, mean and standard deviation (SD) for the normally distributed continuous variables with outliers and median and inter-quartile range (IQR) for continuous variables without outliers. Results are presented in form of tables and graphs.

A sample size of 158 children was used based on calculations from the formula of proportions [20]. The assumption was that with a difference in prevalence of vitamin D deficiency of 20 % between malnourished and non malnourished children, this size would enable us reject the null hypothesis that the vitamin D deficiency for both groups is equal. The study was powered at 0.8 (80 %). The type 1 error probability associated with this test of null hypothesis was 0.05. One hundred seventeen malnourished children were compared with 41 children in the non malnourished group.

Vitamin D, calcium and phosphate statuses were defined based on The Endocrine Society clinical practice guidelines on evaluation, treatment and prevention of vitamin D deficiency by Holick et al. [21]. Vitamin D deficiency, severe vitamin D deficiency, very severe vitamin D deficiency, vitamin D insufficiency and normal vitamin D levels were defined as serum 25(OH)D concentrations ≤20, <10, <5, 21–29 and ≥30 ng/mL. Hypocalcemia, normal serum calcium and hypercalcemia were defined as serum calcium concentration <2.20, 2.20–2.60 and >2.60 mmol/L. Hypophosphatemia, normal phosphate levels and hyperphosphatemia were defined as serum concentration <0.90, 0.90–1.50 and >1.50 mmol/L, respectively.

### Ethical considerations

The study was approved by the Department of Physiology, College of Health Sciences, School of Biomedical Sciences Research and Ethics committee and Mulago Hospital research and ethics committee. Written informed consent was obtained from the children’s parents or guardians prior to enrollment into the study.

## Results

### Socio-demographic, anthropometric and past medical history of the enrolled participants

One hundred fifty-eight children were enrolled, with 117 malnourished and 41 non malnourished. Among the malnourished, 69 (59.0 %) were male and 48 (41 %) female; while 22 (53.7 %) were male and 19 (46.3 %) female in the non malnourished group. In the non malnourished group aged 6–11 months, the median height was 67.5 cm (IQR 61–68 cm); for the children aged 12–17 months, median height was 73.6 cm (IQR 72.4–77.5 cm); for children aged 18–24 months, the median height was 81.5 cm (IQR 74–84 cm); p = 0.341. The median weight for children aged 6–11 months was 7.9 kg (IQR 6.8–8.9 kg); for children 12–17 months, median weight was 10.0 kg (IQR 9.0–11.0 kg); and children 18–24 months, median weight 10.4 kg (IQR 9.5–12.0 kg); p ≤ 0.001).

Among the malnourished, the median height for children aged 6–11 months was 67 cm (IQR 64.5–70 cm); for children aged 12–17 months median height was 70 cm (IQR 68–73.8 cm) and 75 cm (IQR 72–77 cm) for children aged 18–24 months, p = 0.341. The median weight for children 6–11 months was 6 kg (IQR 5.5–7.2 kg); for children 12–17 months, median weight 6.7 kg (IQR 6.0–7.0 kg) and children 18–24 months, median weight 8.0 kg (7.0–8.5 kg), p < 0.001.

Significantly, among the non malnourished children, 5 (12.2 %) were found to have used different unspecified herbal remedies in the 3 months prior to admission, p = 0.012. Of the malnourished children, 58 (49.6 %) had used the herbal remedies prior to hospital presentation. Among the malnourished, 52 (44.4 %) reported a serious/long standing illness in the period of 3 months prior to admission compared to 12 (29.3 %) among the non malnourished, p = 0.027; see Table 1.

**Table 1:** Socio-demographic factors, anthropometric and medical history of the study participants

Characteristic	Non malnourished (n = 41)	Malnourished (n = 117)	Total (n = 158)	P value
Age (months)				
6–11	18 (43.9)	51 (43.6)	69 (43.7)	0.865
12–17	10 (24.4)	33 (28.2)	43 (27.2)	
18–24	13 (31.7)	33 (28.2)	46 (29.1)	
Gender				
Female	19 (46.3)	48 (41.0)	67 (42.4)	0.553
Male	22 (53.7)	69 (59.0)	91 (57.6)	
Median height (IQR) cm				
6–11 months	67.5 (61.0–68.0)	67.0 (64.5–70.0)		0.341
12–17 months	73.6 (72.4–77.5)	70.0 (68.0–73.8)		
18–24 months	81.5 (74.0–84.0)	75.0 (72.0–77.5)		
Median weight (IQR), kg				
6–11 months	7.9 (6.8–8.9)	6.0 (5.5–7.2)		<0.001
12–17 months	10.0 (9.0–11.0)	6.7 (6.0–7.0)		
18–24 months	10.4 (95.0–12.0)	8.0 (7.0–8.5)		
Long standing illness in past 3 months				
Yes	12 (29.3)	52 (44.4)	64 (44.8)	0.027
No	28 (68.3)	51 (43.6)	79 (55.2)	
Feeding type				
Exclusive breast feeding	4 (9.8)	3 (2.6)	7 (4.4)	0.131
Complimentary feeding	19 (46.3)	52 (44.4)	71 (44.9)	
Only solid foods	18 (43.9)	62 (53.0)	80 (50.6)	

There were 117 malnourished and 41 non malnourished children. Twenty-two (53.7 %) of the non malnourished were male and 69 (59.0 %) of the malnourished were male. Non malnourished children aged 6–11 months had a median height of 67.5 cm (61.0–68.0) and a median weight of 7.9 kg (6.8–8.9). There were 52 (44.4 %) of the malnourished who were reported to have a history of a long standing illness in the 3 months prior to admission

### Vitamin D deficiency, calcium, phosphate and sunshine exposure

Among the malnourished children, 51 (43.6 %) were found to have suboptimal vitamin D levels. Thirty-four (29.1 %), 14 (12.0 %) and 3 (2.6 %) were found to have vitamin D insufficiency, deficiency and severe deficiency, respectively. Among the non malnourished, 15 (36.6 %) had suboptimal vitamin D levels, with 8 (19.5 %), 6 (14.6 %) and 1 (2.4 %) found to have insufficiency, deficiency and severe deficiency, respectively.

The mean vitamin D levels were 32.5 ± 12.0 ng/mL among the malnourished and 32.2 ± 10.9 ng/mL among the non malnourished, p = 0.868. The mean calcium levels were 2.0 ± 0.5 mmol/L among the malnourished and 1.7 ± 0.6 mmol/L among the non-malnourished, p = 0.005. The mean phosphate levels were 2.6 ± 1.9 mmol/L among the malnourished and 5.7 ± 2.7 mmol/L among the non-malnourished, p < 0.001.

A significant proportion of children with malnutrition 61 (52.1 %) had normal calcium levels compared to 13 (31.7 %) of the non-malnourished children, p < 0.05. One (2.4 %) of the non-malnourished children had normal phosphate level while 51 (43.6 %) of the malnourished children had normal phosphate levels. Forty (97.6 %) of the non malnourished children had elevated serum phosphate levels compared to 58 (49.6 %) of the malnourished, p = 0.000; Table 2.

**Table 2:** Distribution of serum vitamin D levels, calcium and phosphate among the enrolled children

Characteristic	Non malnourished n = 41 (%)	Malnourished n = 117 (%)	Total n = 158 (%)	P value
Vitamin D (ng/mL)				
<5	0 (0)	0 (0)	0 (0)	0.689
<10	1 (2.4)	3 (2.6)	4 (2.5)	
<20	6 (14.6)	14 (12.0)	20 (12.7)	
21–29	8 (19.5)	34 (29.1)	42 (26.6)	
≥30	26 (63.4)	66 (56.4)	92 (58.2)	
Calcium (mmol/L)				
<2.20	26 (63.4)	50 (43.5)	76 (48.1)	0.063
2.20–2.60	13 (31.7)	61 (52.1)	74 (46.8)	
>2.60	1 (2.5)	4 (3.5)	5 (3.2)	
Phosphate (mmol/L)				
<0.90	0 (0.0)	7 (6.0)	7 (4.4)	0.000
0.90–1.50	1 (2.4)	51 (43.6)	52 (32.9)	
>1.50	40 (97.6)	58 (49.6)	98 (62.0)	

Twenty-six (63.4 %) of the non malnourished children and 66 (56.4 %) of the malnourished had normal serum vitamin D levels. Twenty-six (63.4 %) of the non malnourished and 50 (43.5 %) had low serum calcium levels. Forty (97.6 %) of the non malnourished and 58 (49.6 %) of the malnourished had elevated serum phosphate levels

Among the malnourished children, the median (IQR) serum vitamin D levels was 31 (IQR 9.0, 42.0) ng/mL for those who spent <2 h in the sunshine. There was one outlier in this group with median serum vitamin levels of 64 ng/mL. The median levels among those who spent >4 h exposed to sunshine per day was 34 (IQR 10, 61.0) ng/mL.

Malnourished children were admitted with different infections. N = 104 malnourished children had been admitted with more than 1 diagnosis. These included pneumonia (n = 54), diarrhea (n = 41), measles (n = 15), septicemia (n = 11), malaria (n = 10), Immunosuppression syndrome (n = 6), meningitis (n = 6), anemia (n = 4), sickle cell disease (n = 4) and cerebral palsy (n = 3).

**Fig. 1:**
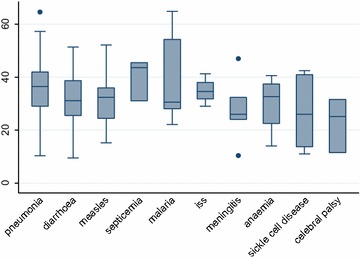
*Box plot* of the admission diagnoses and vitamin D levels amongst the malnourished children. The median (IQR) serum vitamin D level was 36.5 (IQR 29, 42) ng/mL among children admitted with pneumonia. The mean (SD) was 36.3 ± 11.8 ng/mL. There were three outlier; one child admitted with pneumonia with elevated vitamin D levels of >60 ng/mL, one child admitted with meningitis with levels >40 ng/mL and one with serum vitamin D levels <20 ng/mL

Among those admitted with pneumonia, the median (IQR) serum vitamin D levels were 36.5 (IQR 29, 42) ng/mL with a mean (SD) of 36.3 ± 11.8 ng/mL. Children with meningitis had a median (IQR) vitamin levels of 26.0 (24.0, 32.4) ng/mL, cerebral palsy 25.1 (11.5, 31.6) ng/mL, diarrhea 31.1 (25.5, 38.7) ng/mL, measles 32.4 (24.4, 36), septicemia 43.6 (31, 45.5) ng/mL, malaria 30.6 (28, 54.3) ng/mL and sickle cell disease 26.0 (13.7, 41.0) ng/mL. There were three outliers. One child admitted with pneumonia had serum levels >60 ng/mL, one child among those with meningitis had serum levels >40 ng/mL and another had serum vitamin D levels <20 ng/mL, see Fig. 1.

## Discussion

The high prevalence of suboptimal vitamin D levels noted among the malnourished children could probably be attributed to nutrition habits, as the majority, 62 (53.0 %) of the children in this group were no longer breastfeeding but only fed on solid foods. In comparison, a lower proportion, 18 (43.9 %), of the well nourished children were feeding only on solid foods. The majority of the non-malnourished children, 19 (46.3 %) were fed on both solid foods and breast milk. Furthermore, in this study we observed that 58 (49.6 %) of the children in the malnourished group were reported to have used various herbal remedies to treat a number of ailments in the 3 months prior to hospital admission. This could also be one of the contributing factors to the high deficiency prevalence, given the possible interactions between the unquantified and undefined compositions of the herbal formulations.

The suboptimal vitamin D levels in the malnourished group may as well be explained by the illnesses that the children had when they presented to hospital. Children who were found to suffer from central nervous system related infections (meningitis and cerebral palsy) had lower serum vitamin D levels. This may be related to the gross impairment related to these infections that usually will compromise any oral intact. Complications like vomiting associated with these infections will worsen the intake. These findings are comparable with those from other studies that have been carried out among various age groups and patient populations in Mulago hospital and the rest of Africa [22, 23]. There is a wide variation between the prevalence levels of the deficiency in the continent. Studies that have assessed vitamin D suggest a range of status from deficiency (in the rachitic range) to relatively high values. Africa is not a homogeneous entity with respect to geography, climate, water sources, food production and availability, or the religious and cultural practices, skin pigmentation and burden of infectious and chronic disease of its people [1].

The low vitamin D levels among both groups may be responsible for the low calcium levels that were observed. Vitamin D promotes high serum concentrations of phosphate and calcium [24]. Vitamin D induces the synthesis of calcium binding protein, which plays a role in the intestinal absorption of calcium [25]. Vitamin D is responsible for the absorption of calcium from the ileum and reabsorption in the kidneys [25]. High serum phosphate concentrations in both these groups could have contributed to the low calcium levels as it may lead to precipitation of insoluble calcium phosphate in both intracellular and extracellular compartments causing hypocalcemia [26].

Calcium plays a role in the metabolism of phosphate by forming a solubility product that promotes bone mineralization [24]. The high prevalence of low serum calcium levels in the non malnourished children 26 (63.4 %) may be responsible for the high serum phosphate levels 40 (97.6 %). Long standing low dietary calcium intake may result in low serum levels. This calcium imbalance may interfere with the normal serum phosphate concentrations due to the compensatory mechanisms by the parathyroid gland, leading to elevated serum phosphate levels. Vitamin D promotes secondary intestinal phosphate absorption. Vitamin D also promotes proximal renal tubular phosphate reabsorption, reducing urinary loss [24]. The high serum phosphate concentrations among the malnourished children, 58 (49.6 %) can be explained by the prevalence of vitamin D deficiency in this group, 51 (43.6 %).

The mean daily exposure to sunshine among the non-malnourished children was 5.8 h compared to 4.5 h among the malnourished children. This could probably explain the difference in the vitamin D deficiency prevalence levels between these groups. Factors that include the illnesses among the malnourished children may play a role in these sick children spending time indoors, as a larger proportion of the malnourished children 52 (44.4 %) were found to have suffered from a long standing illness in the 3 months period prior to admission. In comparison, 12 (29.3 %) of the well nourished children were reported to have suffered from a significant or long standing illness. This duration of sunshine exposure may play a significant role in the prevalence of vitamin D deficiency observed in the group studied. Compared to related studies done elsewhere in Africa, a higher vitamin D deficiency prevalence has been reported in studies done in South Africa [23], however the difference in the geographical location of these places may be contributory to the difference, as well as the seasonal variations [22]. Cape Town is located on latitude 33°55′S while Kampala is near the equator with sunshine in abundance the whole year round [22]. The factors that affect and may explain the difference in the prevalence are mainly due to differences in the ambient UVB radiation. Stratospheric ozone levels, cloud cover, latitude, season and lower atmospheric pollution are responsible. The factors that affect an individual’s UVB exposure and biological effects of UVB include sun-seeking and sun protective behaviors, skin pigmentation, cultural dress and behavior [27]. Different skin pigmentations are thought to have developed to protect against high ambient UVR whereby those inhabiting low latitudes with high UVR have darker pigmentation to protect against deleterious effects, whilst inhabiting higher latitudes developed fair skin to maximize vitamin D production from much lower ambient UVR [1].

Based on the prevalence of this vitamin deficiency in both groups of the malnourished and non malnourished children, there is need for all children that present to hospitals and clinics to be assessed for this nutrient. Since this vitamin deficiency is highly prevalent among children presenting to hospitals in this region that is rich with sun shine all year round, the possibility of under reporting or under diagnosing this problem among clinicians is high. Vitamin D deficiency often goes undiagnosed or misdiagnosed [28] as fibromyalgia. Symptomatic hypocalcemia is another important, but under-recognized feature [29] among other diseases or complications. One of the reasons is that it is believed that either exposure to sunlight or dietary intake of vitamin D is adequate [28] in this region. Also physicians who perform routine blood work-ups often may obtain a blood calcium value and if it’s found to be normal, they erroneously assume the patient is vitamin D sufficient. Physicians may also wrongly order an analysis for the active form of vitamin D, 1,25-dihydroxyvitamin D [1,25(OH)_2_D], to determine the vitamin D status of the patient. Unfortunately, this is not a measure of vitamin D status and can mislead the physician into thinking that the patient is vitamin D sufficient. Yet, as the person becomes vitamin D-deficient, there is an increase in the concentration of parathyroid hormone (PTH), which increases the renal production of 1,25(OH)_2_D, the circulating concentrations of which become normal or even elevated [28]. These findings indicate a risk group and proposes a ‘critical window’ or group that should be monitored, not only for the emergence of skeletal disorders (rickets, osteomalacia, osteoporosis, fractures) [30] but should also be screened even when asymptomatic. Care should be taken by clinicians not to iatrogenically induce the deficiency, as chronic drug use may block vitamin D [30]. For example, the antimycotic agent ketoconazole is known to inhibit several cytochrome P450-dependent enzymes involved in the biosynthesis of steroid hormones from cholesterol. Since vitamin D is also a sterol synthesized by cytochrome P450-dependent enzymes, inhibition may lead to low vitamin D levels [31]. As well, due to the deficiency, it is largely indicated that national policies and guidelines to actively screen for children with this deficiency may need to be instituted [32].

### Study strengths

This is one of the first descriptive studies of vitamin D in this patient population to be carried out. The study had normal controls that were not known to suffer from any complications or diseases that would interfere with vitamin D metabolism.

### Study limitations

The method used for vitamin D estimation was the electro-chemilumniscence immunoassay which has been shown to underestimate or overestimate vitamin D concentrations. The liquid chromatography-tandem mass spectrometry method of vitamin D estimation that is regarded as the most sensitive and accurate is not available locally [33].

We were unable to assay intact parathyroid hormone and alkaline phosphatase. Long term and/or severe vitamin D deficiency may induce secondary hyperparathyroidism. This will affect serum calcium and phosphate levels.

This was a cross sectional study. The temporal relationship between vitamin D deficiency and malnutrition can not readily be established. We did not have similar numbers of children enrolled in both the malnourished and non malnourished groups.

This was a hospital based study and this may introduce a selection bias. The malnourished children had presented to hospital with underlying complications or problems that may contribute to this vitamin deficiency. We were unable to exclude some causes of vitamin D deficiency like nephritic syndrome, chronic pancreatitis and other causes of malabsorption syndrome among the study participants. However, no study participant had clinical features suggestive of these disorders. Data on the quality and intensity of the sun light on the different days that the children were exposed to is not readily available in this country.

## Conclusions and recommendations

Vitamin D deficiency was found among both the malnourished and non malnourished children. The mean serum vitamin D levels in both the malnourished and non malnourished children were found to be within the normal range. The mean serum calcium levels in both the malnourished and non malnourished were suboptimal. The mean serum phosphate levels were elevated among the malnourished and non malnourished children. Non malnourished children had longer periods of exposure to sunshine.

A wider survey of children in the communities is needed to assess the magnitude of this problem, as this was a hospital-based study. Clinicians should actively screen all children presenting to hospital for serum vitamin d levels regardless of their nutritional status. Parents/guardians should be encouraged to allow their children spend time outdoors in the sun. The ministry of health should put in place programs to widely disseminate information to the public about vitamin D, its role in the growth and development of children and how to avoid the deficiency.

## Abbreviations

DHQ: diet history questionnaire
IQR: interquartile range
MAM: moderate acute malnutrition
NIH: National Institutes of Health
PEM: protein energy malnutrition
PTH: parathyroid hormone
SAM: severe acute malnutrition
SD: standard deviation
UV: ultraviolet
UVR: ultraviolet radiation
UNICEF: United Nations International Children’s Emergency Fund
1,25(OH)_2_D: 1,25-dihydroxyvitamin D
25(OH)D: 25-hydroxyvitamin D
